# Amphotericin B tissue penetration and pharmacokinetics in healthy and *Candida albicans*-infected rats: insights from microdialysis and population modeling

**DOI:** 10.3389/fphar.2024.1515462

**Published:** 2025-01-10

**Authors:** Valdeene Vieira Santos, Laiz Campos Pereira, Jackeline Marley Santos de Araújo, Matheus Antônio da Hora Borges, Carolina Magalhães Brandão, Luisa Oliveira Santos, Cristiane Flora Villarreal, Francine Johansson Azeredo

**Affiliations:** ^1^ Pharmacy Graduate Program, College of Pharmacy, Federal University of Bahia, Salvador, Brazil; ^2^ Laboratory of Pharmacokinetics and Pharmacometrics, Faculty of Pharmacy, Federal University of Bahia, Salvador, Brazil; ^3^ Center for Pharmacometrics and System Pharmacology, College of Pharmacy, University of Florida, Orlando, FL, United States

**Keywords:** Amphotericin B, *Candida albicans*, population pharmacokinetic modeling, microdialysis, rats

## Abstract

**Introduction:**

This study evaluated the relationship between total plasma and free kidney concentrations of amphotericin B (AmB) in healthy and *C. albicans*-infected Wistar rats using microdialysis and has the potential to significantly impact future research in this field and promote the development of antifungal drugs. The findings of this study, which show that plasma levels are a good predictor for AmB kidney concentrations and can be used to optimize its dosing regimen, underscore the importance of this research.

**Methods:**

Microdialysis probe recovery rates were determined by dialysis and retrodialysis *in vitro*, as well as by retrodialysis *in vivo*. The intravenous (i.v.) administration of 2.5 × 10^6^ CFU/mL of *C. albicans* ATCC induced the infection. A 2.5 mg/kg i.v. bolus was used in healthy and *C. albicans*-infected rats (n = 6/group). Plasma and microdialysate samples were analyzed using HPLC-diode-array detection. AmB tissue penetration was analyzed using the ratio between the total plasma and kidney concentrations and population pharmacokinetics (PopPK) to assess the impact of the infection on the pharmacokinetic parameters. The chosen flow rate was set to 1.5 μL/min, and there was no statistical difference between the relative recovery values when changing AmB concentrations.

**Results and Discussion:**

The *in vivo* relative recovery was determined to be 10.9% ± 3.7%. The antifungal tissue penetration was 0.77 and 0.71 for the healthy and infected animals, respectively. The structural PK model with two compartments and linear elimination describes the concentration versus time profile of AmB simultaneously in the plasma and tissue. Infection by *C. albicans* does not interfere with AmB kidney penetration. AmB protein binding is demonstrated to be nonlinear and dependent on the AmB concentration in the plasma of healthy and infected animals.

## 1 Introduction

A significant increase in the incidence of *Candida*-mediated infections has been observed in the last decade, mainly in susceptible individuals associated with high mortality and morbidity (Katsipoulaki et al., 2024). To be a potentially pathogenic yeast, it has to show a wide range of virulence factors and physical characteristics (Colombo et al., 2013; Mayer et al., 2013). *Candida albicans* is the most prevalent agent causing invasive candidiasis (McCarty et al., 2021), and it is also the most isolated species pertaining to these infections from different anatomical sites (Hiller et al., 2011; Kolaczkowski et al., 2010), affecting organs and tissues, such as the lungs, brain, kidneys, bladder, joints, liver, heart, and eyes (Peixoto et al., 2014), with the kidney being the most affected organ (Balk et al., 1978).

For the treatment of invasive candidiasis, amphotericin B (AmB), a drug representing the polyenic class, has significant importance in clinical practice because of its broad spectrum of activities (Ellis, 2002; Wang et al., 2021). Nevertheless, treatment failure is common in infectious diseases (Tøttrup et al., 2014). Insufficient concentration of the used drug at the infection site is one of the leading causes of pharmacological treatment failure (Lagler and Zeitlinger, 2014). For therapeutic success, drug penetration must be sufficient to achieve concentrations that can eliminate or stop the growth of the microorganisms (Felton et al., 2014; Niemirowicz et al., 2017; Ray et al., 2015).

Most infection sites are extravascular, and pharmacological treatment depends on drug diffusion out of the bloodstream and into the interstitial space and intracellular fluid. The penetration capacity of a drug depends on tissue-related factors, such as perfusion, surface area, vascular characteristics, pH, inflammation, protein composition modification, drug transport through efflux pumps (Felton et al., 2014), drug-related factors, and their physicochemical characteristics such as lipophilicity, molecular size, pKa, and plasma protein binding (Hurtado et al., 2014). For these reasons, the use of plasma drug concentrations to determine pharmacokinetic parameters will only yield satisfactory results if blood levels are an appropriate substitute for the levels at the site of infection (Levison and Levison, 2009; Müller et al., 2004).

In this context, the microdialysis technique has important applications, becoming valuable for free drug sampling in the interstitial fluid distribution study (Lagler and Zeitlinger, 2014; MacVane et al., 2014; Lock et al., 2023) in preclinical and clinical pharmacokinetic studies in various tissues such as the bone (Tøttrup et al., 2014), prostate (Hurtado et al., 2014), brain (Levison and Levison, 2009), pancreas (Liu et al., 2014), and pulmonary epithelium (Rottbøll et al., 2015). In addition, this technique allows the comparison of drug penetration in healthy and infected tissues (Joukhadar et al., 2001; Lock et al., 2023).

To date, no study has been conducted that describes the relationship between the total plasma AmB concentrations and free tissue concentrations using microdialysis. Thus, considering the scenario of invasive candidiasis caused by *C. albicans* and the common use of AmB for its treatment, the importance of a pharmacokinetic study in an *in vivo* experimental model emerges to evaluate the influence of infection on pharmacokinetic parameters and in kidney penetration of AmB to relate whether plasma concentrations can predict drug-free tissue levels in the target tissue of invasive candidiasis, with this relationship being established by a population pharmacokinetic (PopPK) model.

## 2 Materials and methods

### 2.1 Quantification of AmB in rat plasma and microdialysate samples

The quantification of AmB in rat plasma and microdialysate samples was performed by HPLC equipped with a diode array detector (HPLC-DAD).

A method for the quantification of AmB in plasma was adopted and validated according to the guideline RDC 27/2012 of the Agência Nacional de Vigilância Sanitária (ANVISA) (BRASIL, 2012). Briefly, plasma aliquots (100 µL) were precipitated with 100 µL of ZnSO_4_ 0.1 M and 200 µL of cold acetonitrile with 0.036% of trifluoroacetic acid and centrifuged at 13,000 *g* at 8°C for 15 min (Barco et al., 2017). The supernatant was injected into the HPLC-DAD system (Shimadzu CBM-20^a^ system controller, LC-6AD solvent delivery system, SPD-M20A detector, and DGU-20^a^ deaerator) (Shimadzu Corp., Kyoto, Japan). Chromatography was performed by using a Nucleosil^®^ column (150 mm × 4.6 mm internal diameter) (Macherey-Nagel, Dorean) protected by a Uniguard^®^ (Thermo Fisher Scientific) guard column packed with the same material. Analytes were separated by gradient elution, with mobile phase A consisting of acetate buffer pH 4.0 and mobile phase B consisting of acetonitrile at a flow rate of 1 mL/min. The percentage of mobile phase B was maintained at 40% for 4 min and then programmed to reach 80% in 4 min. The column was finally reconditioned at 40% B in 1 min for a total run time of 9 min. Detection was measured at 407 nm (Italia et al., 2009). The analyses were conducted using LC Solution software.

For the quantification of AmB in microdialysate samples, the same chromatographic conditions as for the bioanalytical method were used. The method was validated according to the ANVISA guidelines for analytical methods (RDC 166-2017) (BRASIL, 2017).

The methods were sensitive and accurate within the concentration range of 0.25–10 μg/mL. A linear calibration curve was obtained in this concentration range. The methods were validated by constructing six calibration curves on two separate days and quality control samples (0.4, 1.5, and 6 μg/mL). The intra-assay and inter-assay precision was not more than 5.0%, and the accuracy was between 95% and 105% for the analytical method. The bioanalytical method intra-assay and inter-assay precision and accuracy were not more than 20% for the lower limit of quantification and not more than 15% for the other concentrations.

### 2.2 Induction of infection

Male Wistar rats (250–350 g) were kept in cages in groups of five animals under conditions of controlled temperature (22°C ± 2°C) and humidity (55% ± 5%) and 12-h of light/dark cycles with feed and water provided *ad libitum*. The protocols for animal experiments were approved by the Veterinary School of the Federal University of Bahia (UFBA) Animal Use Ethics Committee (protocol number 26/2018).

The infection protocol for Wistar rats employed in this study was previously described by de Araujo et al. (2009). Briefly, the animals were infected with *C. albicans* (ATCC 10231) 2 days before the pharmacokinetics experiments by the intravenous(i.v.) injection through the lateral tail vein of 0.1 mL of the inoculum containing 2.5 × 10^6^ CFU/mL of the fungal strain, which was prepared in 0.9% sterile saline.

### 2.3 Influence of the perfusion flow rate and drug concentration on *in vitro* microdialysis probe recovery

The microdialysis system consisted of a PHD 2000 Infuse/Withdraw (Harvard Apparatus) microsyringe (diameter 4.61 mm, 1 mL) to provide the perfusate solution, which was attached to a microdialysis probe CMA/20 Elite (membrane length of 4 mm and cutoff of 20 kDa) (CMA/Microdialysis AB, Solna, Sweden).

The influence of the perfusion flow rate on the relative recovery of AmB was evaluated by using three flow rates, 1, 1.5, and 2 μL/min, using a 2 μg/mL solution of AmB. After analyzing the relative recovery of the different flows, the flow rate of 1.5 μL/min was selected for analyzing the influence of different AmB concentrations on relative recovery using three different concentrations to determine the recovery: 2.5, 3, and 4 μg/mL. The retrodialysis and dialysis methods were used to determine the relative recovery of AmB (*n* = 3) in both analyses.

The relative recovery by dialysis (RR_D_) was calculated by Equation 1:

$${RR}_{D}\;\left(\%\right)=\left(\frac{C_{dial}}{C_{ext}}\right)x\;100,$$ (1)

where $C_{dial}$ is the drug concentration in the dialysate and $C_{ext}$ is the drug concentration in the medium surrounding the microdialysis probe.

The relative recovery by retrodialysis (RR_RD_) was calculated using Equation 2:

$${RR}_{RD}\;\left(\%\right)=\left(\frac{C_{perf}-C_{dial}}{C_{perf}}\right)x\;100,$$ (2)

where $C_{perf}$ is the drug concentration in the perfusate solution.

### 2.4 *In vivo* microdialysis probe recovery

On the day of the experiments, the animals were anesthetized with urethane (1.25 g/kg intraperitoneally [IP]). The animals were put in the lateral decubitus position, and the kidney was exposed. The microdialysis probes were inserted into the kidney cortex and were allowed to equilibrate inside the kidney for 1 h before the *in vivo* recovery of the probes was performed. After that, perfusion with Ringer’s solution at a flow rate of 1.5 μL/min was carried out.

AmB microdialysis probe recovery *in vivo* was determined by retrodialysis. Following the kidney exposure procedure described above and after equilibration, Ringer’s solution was replaced by 3 μg/mL (using the 1.5 μL/min flow rate) AmB in Ringer’s solution. Three microdialysate samples were collected from each probe before the animal experiment (n = 6). Drug concentrations in the dialysate sample (C_dial_) and the perfusate solution (C_perf_) were determined by HPLC. The *in vivo* apparent recovery by retrodialysis was calculated using Equation 2.

### 2.5 Population pharmacokinetic

After the microdialysis probes were inserted into the kidney cortex and allowed to equilibrate for 1 h, 2.5 mg/kg i.v. dosing of AmB was administrated to the animals. The AmB administration was prepared in injectable water (Anforicin B^®^, Cristália, Brazil). The rats were randomly assigned to four groups of six animals each: two groups for plasma pharmacokinetics and two groups for tissue penetration, with each group having a group of healthy and *C. albicans*-infected animals.

Microdialysate samples were collected over 20 h at 1-h intervals and assayed directly after the experiment, without processing. Blood samples were collected through the lateral tail vein immediately after dosing at 0.083, 0.25, 0.5, 1, 1.5, 2, 4, 8, 12, 24, and 36 h. Blood samples were harvested into tubes containing heparin and centrifuged after collection. Plasma was separated and stored at −18°C until analysis.

The concentrations of AmB in the kidney were calculated from the measured microdialysate concentrations by using the relative recovery rate determined by retrodialysis *in vivo*. The total area under the concentration–time curve from 0 h to infinity (AUC_0- ∞ _) was calculated by the linear trapezoidal rule for plasma and tissue. The ratio of drug penetration into the kidney was calculated using Equation 3:

$$Ratio\;of\;penetration=\frac{{AUC}_{0-\infty tissue\;}}{{AUC}_{0-\infty plasma\;}\;}.$$ (3)

PopPK was performed using Monolix Suite™ 2024R1 (Lixoft, France). A total of 266 observations (116 plasma and 150 kidney measurements) derived from 12 rats (six healthy and six infected) were included in the data set for PopPK analysis. Plasma and kidney microdialysate samples were simultaneously fitted to the model. Several structural models were proposed to characterize the PK of AmB, including two- and three-compartment models and normal and lognormal distribution.

The inter-individual variability on the fixed-effect model parameters was described by Equation 4 using an exponential model:

$$P_{i}=P_{pop}\times \exp\left(n_{i,p}\right),$$ (4)

where $P_{i}$ represents the parameter estimate for the individual normally distributed, $P_{pop}$ is the typical parameter estimate in the population, and $n_{i,p}$ is the random effect accounting for the individual difference from the typical value normally distributed with a mean of 0 and variance Ω. Correlations between random effects were tested. The residual variability was tested with combined, proportional, and constant error models.

The comparison between hierarchical models was based on graphic and statistical methods, with the reduction of the objective function value approximately equal to −×2 log(likelihood); reduction of the standard error of the estimates; graphical evaluation of the goodness-of-fit (GOF), which included graphs of the predicted individual concentrations *versus* the observed concentrations; evaluation of weighted residuals *versus* population predicted; and visual predictive checks (VPCs, 1,000 simulations). The effect of categorical covariate infection by *C. albicans* on PK parameters was investigated once an adequate model was finalized. The covariate *C. albicans* infection was assessed using the likelihood ratio test and the Wald test (*P* < 0.05), and the precision of parameter estimates and the GOF were found to be improved. The final model was tested by bootstrap (n = 200) using the 95% confidence interval of (CI) 2.5 to 97.5 percentiles for the model parameters.

### 2.6 Protein binding

Two AmB concentrations (2 and 8 μg/mL) based on the total plasma concentrations observed in animals after i.v. administration of 2.5 mg/kg dose were used to evaluate *in vitro* rat plasma protein binding by ultrafiltration. Plasma aliquots from healthy and *C. albicans*-infected rats were placed into the upper part of the Centrifee^®^ ultrafiltration devices (95 mm, 30 kDa cut-off; Millipore Corp.) and centrifuged at 2,000 *g* for 15 min at 25°C ± 1°C. Ultrafiltrates were collected, and the AmB concentration was determined in each sample by HPLC-DAD, as described previously. Triplicates were analyzed for each concentration, and the AmB-free fraction was determined by the ratio between the ultrafiltration and total plasma concentrations.

## 3 Results

The relative recovery rates of AmB determined *in vitro* by the dialysis methodology (RR_D_) were 8.1% ± 4.1%, 22.5% ± 8.5%, and 8.0% ± 3.0%, and those determined by the retrodialysis methodology (RR_RD_) were 12.9% ± 2.3%, 20.0% ± 6.0%, and 9.2% ± 2.7%, for the 1.0, 1.5, and 2.0 μL/min flow rates, respectively. For the same flow rates, no statistically significant difference was observed between the two methods (*P >* 0.05), showing that AmB does not bind to microdialysis tubing and that retrodialysis can be used to determine the relative recovery *in vivo*; the flow rate of 1.5 μL/min is used for subsequent tests as it presents greater relative recovery.

The influence of the drug concentration on the microdialysis recoveries of AmB was evaluated by using a 1.5 μL/min flow rate. The average relative recovery of the analyzed concentrations (2.5, 3.0, and 4.0 μg/mL) obtained by dialysis was 33.1% ± 7.6%, and by retrodialysis, it was 34.5% ± 2.4%. No statistically significant differences (*p* > 0.05) were observed in the values obtained between the methods or between the AmB concentrations tested. The RR_RD_ *in vivo* was determined to be 10.9% ± 3.7%.

AmB protein binding was determined to be 56.4% ± 4.6% for 2 μg/mL and 25.5% ± 3.9% for 8 μg/mL in the plasma of healthy rats, and it was 60.9% ± 12.1% for 2 μg/mL and 41.8% ± 1.7% for 8 μg/mL in the plasma of *C. albicans*-infected rats, demonstrating it to be nonlinear and dependent on the AmB plasma concentration.

**FIGURE 1:**
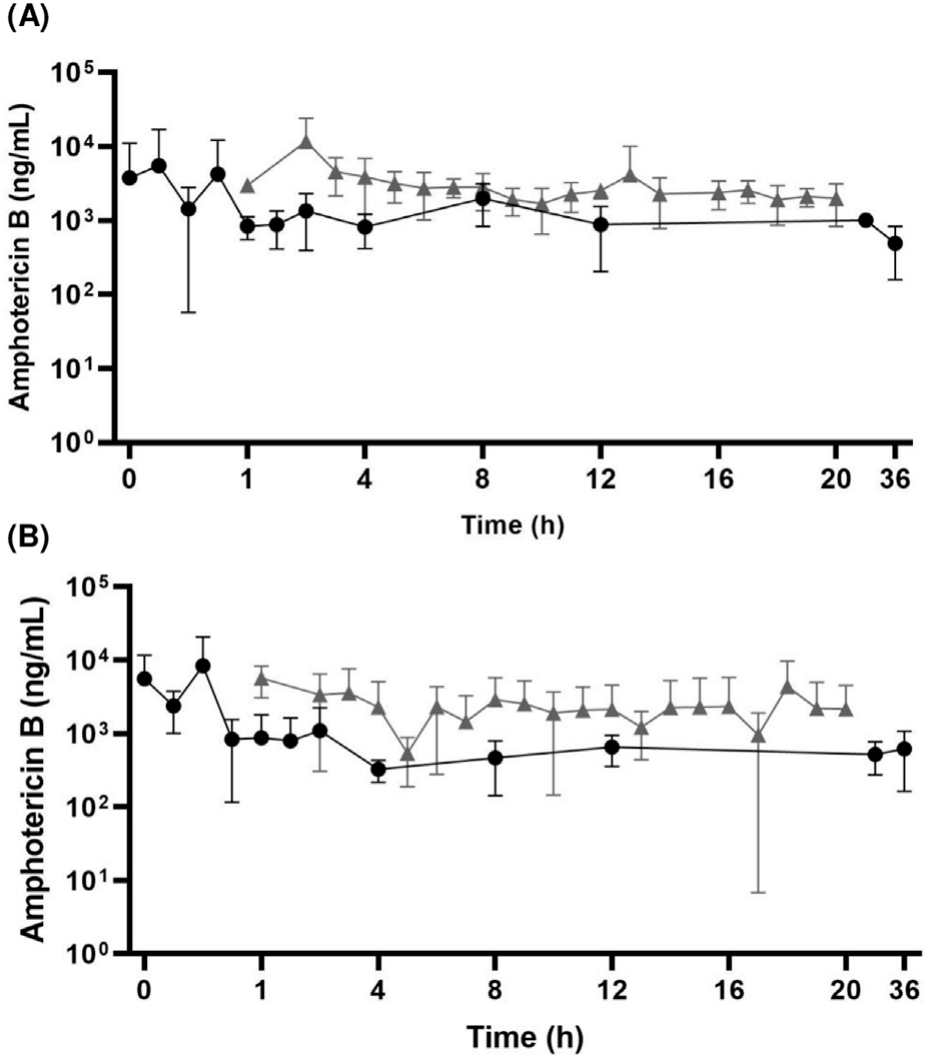
Mean AmB total plasma **(A)** and free kidney **(B)** concentration–time profiles after 2.5 mg/kg i.v. bolus dosing in healthy (●) and *Candida albicans*-infected (▲) Wistar rats (mean ± standard deviations are shown; n = 6/group).

AmB total plasma and free kidney concentrations *versus* time profiles after the administration of a 2.5 mg/kg i.v. dose to study the plasma pharmacokinetics and kidney penetration in healthy and *C. albicans-*infected rats are shown in Figure 1. The AmB kidney penetration was 0.77 in healthy rats and 0.71 in infected rats. The pharmacokinetic parameters showed no statistical difference (*P* > 0.05) for healthy and *C. albicans*-infected animals and between the plasma and kidney. The mean AUC_0- ∞_ was 96.3 ± 54.8 μg h/mL for plasma and 74.1 ± 31.7 μg h/mL for tissue in healthy animals and 59.8 ± 30.1 μg h/mL for plasma and 42.3 ± 36.0 μg h/mL for tissue in *C. albicans*-infected rats.

**FIGURE 2:**
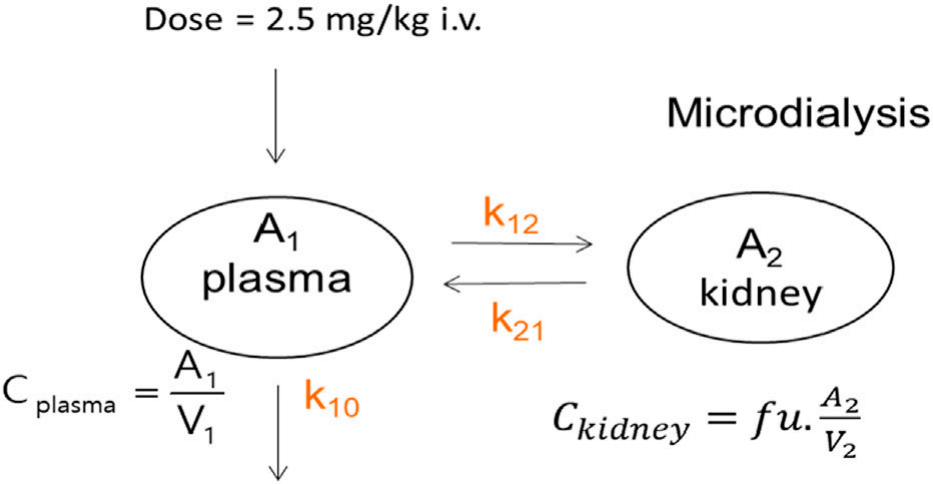
Schematic representation of the PopPK model. The model describes the AmB PK following an i.v. administration using a two-compartment structure. The central compartment (A1) represents the plasma and well-perfused tissues. The peripheral compartment (A2) represents the kidney, where unbound drug concentrations (fu) are of interest. Drug distribution between plasma and the kidney is governed by the rate constants k12 (from plasma to the kidney) and k21 (from the kidney to plasma). The drug is eliminated from the central compartment via first-order elimination (k10).

In the population pharmacokinetic analysis, a two-compartment model with linear elimination for both the plasma total and free kidney concentrations was selected as the final model (Figure 2). The system of differential equations for the final model is given in Equations 5–8:

$$\frac{dA_{1}}{dt}=A_{2}\times k_{21}-A_{1}\times k_{12}-A_{1}\times k_{10}.$$ (5)

$$\frac{dA_{2}}{dt}=0.56\times \left(A_{1}\times k_{12}-A_{2}\times k_{21}\right).$$ (6)

$$C_{plasma\;}=\frac{A_{1}}{V_{1}}.$$ (7)

$$C_{kidney}=\frac{A_{2}}{V_{2}}.$$ (8)

Here, $A_{1}$ and $A_{2}$ are the drug amounts in the central and kidney compartments, respectively, t is time, $k_{12}$ and $k_{21}$ are the first-order distribution rate constants, $C_{plasma}$ is the observed plasma concentration, $C_{kidney}$ is the free concentration measured in the kidney, $V_{1}$ represents the volume of the central compartment, and $V_{2}$ is the volume of distribution in the kidney compartment.

**FIGURE 3:**
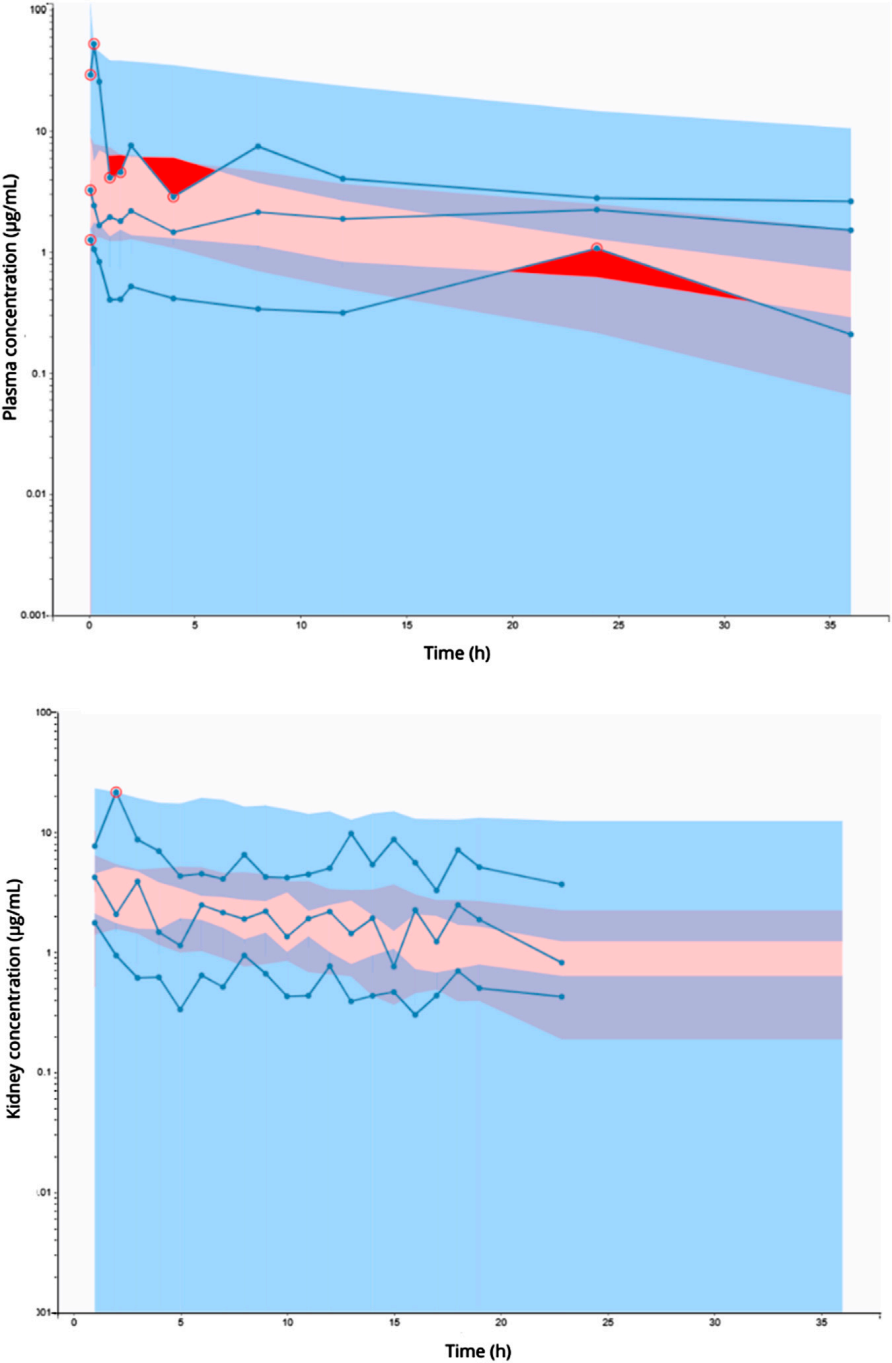
Visual predictive checks of total plasma (upper panel) and free kidney (lower panel) AmB concentrations versus time for the final model. Dots show the observed plasma concentrations. The lines show the fifth percentile (lower dashed blue line), 50th percentile (blue line), and 95th percentile (upper dashed blue line) of the observed plasma concentrations. The shaded areas show the fifth percentile (blue shading), 50th percentile (red shading), and 95th percentile (blue shading) of the observed kidney concentration.

The estimated parameter values are presented in Table 1. The best residual error model for the total plasma and free kidney concentrations was the proportional model, which was based on goodness-of-fit and lower AIC. In the final model, inter-individual variability (IIV) was incorporated in V_1_, V_2_, k_10_, and k_12_. The model validation methods showed predictive performance for the AmB model. The VCPs for total plasma and free kidney concentrations are shown in Figure 3, confirming the adequacy of the model. The VPCs confirmed that the model was adequate to simultaneously describe the data of all the evaluated groups. The categorical covariate infection by *C. albicans* did not influence the model performance (*p* > 0.05). The stability of the final model was assessed by bootstrap analysis, with parameters estimated by the model agreeing with the respective confidence intervals estimated for 200 bootstrap replicates (Table 1).

**TABLE 1:** Parameter estimates in the final AmB population PK model.

Parameter	Estimate [R.S.E. (%)]	Bootstrap (n = 200)		
		P 2.5	Median	P 97.5
V_1_ (mL/kg)	3.3 (46.4)	1.42	3.52	6.95
V_2_ (mL/kg)	328.58 (21.6)	224.48	342.87	533.39
k_10_ (h^-1^)	8.53 (33.4)	3.21	8.07	15.81
k_12_ (h^-1^)	91.58 (31.3)	49.45	90.33	287.5
k_21_ (h^-1^)	1.07 (15.1)	0.68	1.08	2.3

Inter-individual variability						
	Estimate	C.V. (%)	R.S.E. (%)	P 2.5	Median	P 97.5
ω^2^ (V_1_)	0.87	106.18	24.3	0.15	0.79	1.26
ω^2^ (V_2_)	0.67	74.71	23.4	0.29	0.61	0.81
ω^2^ (k_10_)	0.54	58.74	40.6	0.066	0.42	0.99
ω^2^ (k_12_)	0.57	62.34	47.9	0.15	0.52	0.98

Error model parameters				
σ2 (proportional error, plasma)	0.76 (11.3)	0.57	0.77	0.96
σ2 (proportional error, kidney)	0.65 (8.26)	0.53	0.65	0.77

## 4 Discussion

This is the first study that assesses the renal penetration of AmB using microdialysis in healthy and *C. albicans*-infected rats. The sites of candidiasis systemic infections are extravascular, so the pharmacological treatment depends on the diffusion of the antifungal drug out of the bloodstream and into the interstitial space and intracellular fluid (Levison and Levison, 2009). Drug penetration must be sufficient to achieve concentrations that eliminate or stop the growth of the fungus, having therapeutic success, and, ideally, the plasma concentration should reflect tissue concentrations (Felton et al., 2014).

Microdialysis has proven to be a powerful tool to investigate the tissue distribution of antimicrobial agents. Compared with traditional sampling methods, microdialysis is the only technique that allows the collection of the protein unbound drug since the unbound fraction of the drug is available for absorption, distribution, metabolism and elimination, and delivery to the target sites for pharmacodynamic actions. Furthermore, microdialysis allows systemic and local administration of drugs, collection of small-molecular weight substances from the extracellular space, and conducting of the experiment in awake animals because the perfusion of the probe is not painful. Regarding limitations, microdialysis is an invasive procedure, and there are analytical difficulties with some molecules and low membrane recoveries with high-molecular-weight compounds (Parent et al., 2001; Lietsche et al., 2014; Tsai, 2003).

*In vitro* calibration of microdialysis probes is considered an essential step for further *in vivo* studies. Although *in vitro* calibration does not eliminate the need to assess drug recovery *in vivo*, it provides essential information on drug gain and loss and the feasibility of *in vivo* calibration (MacVane et al., 2014). It was possible to define the microdialysis workflow by evaluating the influence of the different test flow rates on the relative recovery of AmB and drug concentration. Among all flow rates tested, the relative recovery of AmB was greater in the flow rate of 1.5 μL/min (the flow being chosen for this work) with RR_D_ 22.5% ± 8.5% and RR_RD_ 20.0% ± 6.0%, with no statistical difference between rates of recovery determined by the two methods used.

There is a change in recovery for different flow rates. In general, lower flow rates result in higher relative recovery values, and high flow rates result in lower recovery rates (Plock and Kloft, 2005). The flow rate of 1 μL/min showed less relative recovery when compared to the flow rate of 1.5 μL/min. This phenomenon can occur in perfusion flows below or equal to 1 μL/min due to membrane adsorption processes (Kjellström et al., 1998). Low flow rates also increase the collection interval due to the sample volume and the quantification limit of the analytical method. On the other hand, high flow rates can be conducive to the ultrafiltration process due to the pressure accumulated in the microdialysis tube, resulting in liquid flow out of the probe (Plock and Kloft, 2005), as was the case with the 2 μL/min flow rate, which had a less relative recovery.

In assessing the influence of AmB concentration on recovery from microdialysis, the results showed that there was no statistical difference in the relative recovery among the concentrations and methods evaluated, showing that the recovery of AmB is independent of the investigated concentration range and will not change in concentrations in floating tissue.

The RR_RD_ *in vivo* was lower and statistically different from the recovery determined *in vitro* by retrodialysis at the same rate. This phenomenon is due in large part to the reduced volume of fluid and the increase in tissue tortuosity, with an increase in the diffusion length caused by the impediment imposed by cellular structures as to the connectivity of spaces. In addition, tissue diffusion can be further delayed by binding the analyte to cell surface proteins along the diffusion path, indicating that the *in vivo* recovery of an analyte must be less than the *in vitro* recovery of the same analyte in a solution (Shippenberg and Thompson, 2001), as previously reported for other drugs (Torres et al., 2017).

AmB total plasma and free kidney concentration–time profiles were obtained after the administration of 2.5 mg/kg dose. This dose, still in AmB linearity (Walsh et al., 1998), was used in the pharmacokinetic study due to the low *in vivo* relative recovery of AmB and the quantification limit of the validated analytical method. After the administration of this dose, no change was observed in the pharmacokinetic parameters and renal distribution of AmB during *C. albicans* infection in rats using a bi-compartmental model with kidney/plasma redistribution and linear elimination. The final model adequately describes the data with adequate diagnostic parameters and graphics. Observations *versus* model predictions, GOF, and VPC plots (Figure 3) support the adequacy of the PK model in describing the experimental data.

Some studies demonstrate that an infection can alter the physiological characteristics of the infected tissue due to the inflammatory process developed by the host to eliminate the invasive organism, as observed in soft tissue infections (Joukhadar et al., 2001), septic shock (Joukhadar et al., 2002), or infections (Tegeder et al., 2002) with several antibiotics. Moxifloxacin (Joukhadar et al., 2003), imipenem (Tegeder et al., 2002), piperacillin, and cefpirome (Joukhadar et al., 2002) had less penetration into inflamed, infected tissues, and critical volunteers. There is no clear possible explanation for the poor distribution of tissues and organs. It is expected that the local inflammation itself increases capillary permeability, but studies suggest that because of the systemic inflammatory response of the infection, there is capillary leakage, generating interstitial edema. Likewise, other studies indicate that an infection, mainly caused by *Candida* sp, does not interfere with the renal penetration of drugs, as with voriconazole (de Araujo et al., 2009) and fluconazole (Azeredo et al., 2012), and therefore, free kidney concentration is predicted by total plasma concentration. Thus, it is suggested that the phenomenon observed with AmB in this study is not exclusive to this drug but is probably shared by other anti-infective agents.

AmB concentrations decreased rapidly during the first 4 h after administration and remained almost constant until 36 h, revealing a phase of slow elimination, as previously reported in Fielding et al., 1991; Fielding et al., 1991. Studies using microdialysis to determine the free tissue concentrations of AmB were not found in the literature. Population parameters showed a considerable tissue distribution of AmB, with V_2_ of 328.58 mL/kg and k_12_ 91.58 h^-1^. The entire free concentration of AmB in the plasma can be distributed to the kidneys.

AmB protein binding determined for two plasma concentrations (2 and 8 μg/mL) *in vitro* using both healthy and *C. albicans*-infected rat plasma demonstrated that protein binding was concentration-dependent. In the literature, there are no data on the connection to plasma proteins of AmB in rats, although this drug has been used and studied for a long time. The human data demonstrate that AmB is mainly bound to plasma lipoproteins (Brajtburg et al., 1984) and also human serum albumin and human alfa-1-acid glycoprotein (Bekersky et al., 2002); Bekersky et al. (2002) evaluated the *in vitro* binding of AmB to human plasma using increasing concentrations of drugs. AmB was shown to be distinctly nonlinear and dependent on the concentration, which is not expected for most drugs due to the saturation of the binding site with increased plasma drug concentration (Bekersky et al., 2002). Nonlinear protein binding has been studied and discussed by several authors after some tetracyclines presented this type of behavior and showed the importance of the exposure and penetration of the drug in the tissues (Deitchman et al., 2018). Still, regarding the results of AmB binding to proteins, there was no difference in the binding of the plasma of healthy and *C. albicans-*infected rats. The concentration dependency in our study did not interfere drastically since the plasma concentrations of the drug only reached the concentration of 8 μg/mL in a few moments of the experiment, as can be seen in the concentration *versus* time curve (Figure 1).

In conclusion, a population PK model capable of simultaneously describing plasma and kidney concentrations of AmB that was generated and estimating the parameters associated with tissue distribution in the rats’ model, demonstrating that there is no statistical difference in drug penetration in the kidneys in healthy and *C. albicans*-infected groups. In addition, the data from the present study generate the hypothesis that AmB has a nonlinear protein binding. Therefore, infection by *C. albicans* does not interfere with kidney penetration of AmB, which easily penetrates, and plasma AmB concentrations are considered a good predictor for free kidney concentrations and can be used to optimize AmB regimens to treat disseminated candidiasis considering the drug–protein binding.

## Data Availability

The raw data supporting the conclusions of this article will be made available by the authors, without undue reservation.

## References

[B1] Azeredo F. J. Verlindo de Araújo B. Haas S. E. Torres B. Pigatto M. Andrade C. de (2012). Comparison of fluconazole renal penetration levels in healthy and Candida albicans-infected wistar rats. Antimicrob. Agents Chemother. 56 (11), 5852–5857. 10.1128/AAC.01323-12 22948869 PMC3486536

[B2] Balk M. W. Crumrine M. H. Fischer G. W. (1978). Evaluation of miconazole therapy in experimental disseminated candidiasis in laboratory rats. Antimicrob. Agents Chemother. 13 (2), 321–325. 10.1128/AAC.13.2.321 646350 PMC352234

[B3] Barco S. Zunino A. D’Avolio A. Barbagallo L. Maffia A. Tripodi G. (2017). A rapid and robust UHPLC-DAD method for the quantification of amphotericin B in human plasma. J. Pharm. Biomed. Analysis 138, 142–145. 10.1016/j.jpba.2017.01.048 28199895

[B4] Bekersky I. Fielding R. M. Dressler D. E. Lee J. W. Buell D. N. Walsh T. J. (2002). Plasma protein binding of amphotericin B and pharmacokinetics of bound versus unbound amphotericin B after administration of intravenous liposomal amphotericin B (AmBisome) and amphotericin B deoxycholate. Antimicrob. Agents Chemother. 46 (3), 834–840. 10.1128/AAC.46.3.834-840.2002 11850269 PMC127463

[B5] Brajtburg J. Elberg S. Bolard J. Kobayashi G. S. Levy R. A. Ostlund R. E. (1984). Interaction of plasma proteins and lipoproteins with amphotericin B. J. Infect. Dis. 149 (6), 986–997. 10.1093/infdis/149.6.986 6376657

[B6] BRASIL (2012). RDC No 27, de 17 de Maio de 2012. Available at: http://bvsms.saude.gov.br/bvs/saudelegis/anvisa/2012/rdc0027_17_05_2012.html.

[B7] BRASIL (2017). RDC No 166, de 24 de julho de 2017. Available at: https://www.in.gov.br/materia.

[B8] Colombo A. L. Guimarães T. Camargo L. F. A. Richtmann R. Queiroz-Telles F. de Salles M. J. C. (2013). Brazilian Guidelines for the Management of Candidiasis - a Joint Meeting Report of Three Medical Societies: Sociedade Brasileira de Infectologia, Sociedade Paulista de Infectologia and Sociedade Brasileira de Medicina Tropical. Braz. J. Infect. Dis. 17, 283–312. 10.1016/j.bjid.2013.02.001 23693017 PMC9427385

[B9] de Araujo B. V. Farias da Silva C. Haas S. E. Costa T. D. (2009). Free renal levels of voriconazole determined by microdialysis in healthy and Candida sp.-infected wistar rats. Int. J. Antimicrob. Agents 33 (2), 154–159. 10.1016/j.ijantimicag.2008.08.020 19010646

[B10] Deitchman A. N. Singh R. S. P. Derendorf H. (2018). Nonlinear protein binding: not what you think. J. Pharm. Sci. 107 (7), 1754–1760. 10.1016/j.xphs.2018.03.023 29626534 PMC6042850

[B11] Ellis D. (2002). Amphotericin B: spectrum and resistance. J. Antimicrob. Chemother. 49, 7–10. 10.1093/jac/49.suppl_1.7 11801575

[B12] Felton T. Troke P. F. Hope W. W. (2014). Tissue penetration of antifungal agents. Clin. Microbiol. Rev. 27 (1), 68–88. 10.1128/CMR.00046-13 24396137 PMC3910906

[B13] Fielding R. M. Smith P. C. Wang L. H. Porter J. Guo L. S. (1991). Comparative pharmacokinetics of amphotericin B after administration of a novel colloidal delivery system, ABCD, and a conventional formulation to rats. Antimicrob. Agents Chemother. 35 (6), 1208–1213. 10.1128/AAC.35.6.1208 1929263 PMC284312

[B14] Hiller E. Zavrel M. Hauser N. Sohn K. Burger-Kentischer A. Lemuth K. (2011). Adaptation, adhesion and invasion during interaction of Candida albicans with the host--focus on the function of cell wall proteins. Int. J. Med. Microbiol. IJMM 301 (5), 384–389. 10.1016/j.ijmm.2011.04.004 21571590

[B15] Hurtado F. K. Weber B. Derendorf H. Hochhaus G. Costa T. D. (2014). Population pharmacokinetic modeling of the unbound levofloxacin concentrations in rat plasma and prostate tissue measured by microdialysis. Antimicrob. Agents Chemother. 58 (2), 678–686. 10.1128/AAC.01884-13 24217697 PMC3910848

[B16] Italia J. L. Singh D. Ravi Kumar M. N. V. (2009). High-performance liquid chromatographic analysis of amphotericin B in rat plasma using alpha-naphthol as an internal standard. Anal. Chim. Acta 634 (1), 110–114. 10.1016/j.aca.2008.12.006 19154818

[B17] Joukhadar C. Frossard M. Mayer B. X. Brunner M. Klein N. Siostrzonek P. (2001). Impaired target site penetration of beta-lactams may account for therapeutic failure in patients with septic shock. Crit. Care Med. 29 (2), 385–391. 10.1097/00003246-200102000-00030 11246321

[B18] Joukhadar C. Klein N. Mayer B. X. Kreischitz N. Delle-Karth G. Palkovits P. (2002). Plasma and tissue pharmacokinetics of cefpirome in patients with sepsis. Crit. Care Med. 30 (7), 1478–1482. 10.1097/00003246-200207000-00013 12130965

[B19] Joukhadar C. Stass H. Müller-Zellenberg U. Lackner E. Kovar F. Minar E. (2003). Penetration of moxifloxacin into healthy and inflamed subcutaneous adipose tissues in humans. Antimicrob. Agents Chemother. 47, 3099–3103. 10.1128/AAC.47.10.3099-3103.2003 14506015 PMC201117

[B20] Katsipoulaki M. Stappers M. H. T. Malavia-Jones D. Brunke S. Hube B. Gow N. A. R. (2024). Candida albicans and Candida glabrata: global priority pathogens. Microbiol. Mol. Biol. Rev. MMBR 88 (2), e0002123. 10.1128/mmbr.00021-23 38832801 PMC11332356

[B21] Kjellström S. Emnéus J. Laurell T. Heintz L. Marko-Varga G. (1998). On-line coupling of microdialysis sampling with liquid chromatography for the determination of peptide and non-peptide leukotrienes. J. Chromatogr. A 823 (1), 489–496. 10.1016/S0021-9673(98)00361-6 9818423

[B22] Kolaczkowski M. Kolaczkowska A. Sroda K. Ramalhete C. Michalak K. Mulhovo S. (2010). Substrates and modulators of the multidrug transporter Cdr1p of Candida albicans in antifungal extracts of medicinal plants. Mycoses 53 (4), 305–310. 10.1111/j.1439-0507.2009.01711.x 19460101

[B23] Lagler H. Zeitlinger M. (2014). Tissue penetration of antibiotics. Does the treatment reach the target site? Med. Klin. Intensivmed. Und Notfallmedizin 109 (3), 175–181. 10.1007/s00063-013-0309-0 24691884

[B24] Levison M. E. Levison J. H. (2009). Pharmacokinetics and pharmacodynamics of antibacterial agents. Infect. Dis. Clin. N. Am. 23 (4), 791–815. 10.1016/j.idc.2009.06.008 PMC3675903 19909885

[B25] Lietsche J. Gorka J. Hardt S. Karas M. Klein J. (2014). Self-built microdialysis probes with improved recoveries of ATP and neuropeptides. J. Neurosci. Methods 237, 1–8. 10.1016/j.jneumeth.2014.08.015 25172804

[B26] Liu D. Xu S. Xiao H. Wang Z. Mao N. Zhou J. (2014). Quantitative determination of unbound levofloxacin by simultaneous microdialysis in rat pancreas after intravenous and oral doses. J. Pharm. Pharmacol. 66 (9), 1215–1221. 10.1111/jphp.12252 24961375

[B27] Lock G.De A. Helfer V. E. Dias B. B. Torres B. G. S. Araújo B. V.De Costa T. D. (2023). Population pharmacokinetic modeling of the influence of chronic and acute biofilm-forming Pseudomonas aeruginosa lung infection on ciprofloxacin free pulmonary and epithelial lining fluid concentrations. Eur. J. Pharm. Sci. 189, 106546. 10.1016/j.ejps.2023.106546 37517670

[B28] MacVane S. H. Housman S. T. Nicolau D. P. (2014). *In vitro* microdialysis membrane efficiency of broad-spectrum antibiotics in combination and alone. Clin. Pharmacol. Adv. Appl. 6, 97–101. 10.2147/CPAA.S65389 PMC4051625 24940084

[B29] Mayer F. L. Wilson D. Hube B. (2013). Candida albicans pathogenicity mechanisms. Virulence 4 (2), 119–128. 10.4161/viru.22913 23302789 PMC3654610

[B30] McCarty T. P. White C. M. Pappas P. G. (2021). Candidemia and invasive candidiasis. Infect. Dis. Clin. N. Am. 35 (2), 389–413. 10.1016/j.idc.2021.03.007 34016283

[B31] Müller M. Peña A. d. Derendorf H. (2004). Issues in pharmacokinetics and pharmacodynamics of anti-infective agents: distribution in tissue. Antimicrob. Agents Chemother. 48 (5), 1441–1453. 10.1128/AAC.48.5.1441-1453.2004 15105091 PMC400530

[B32] Niemirowicz K. Durnaś B. Piktel E. Bucki R. (2017). Development of antifungal therapies using nanomaterials. Nanomedicine Lond. Engl. 12 (15), 1891–1905. 10.2217/nnm-2017-0052 28703684

[B33] Parent M. Bush D. Rauw G. Master S. Vaccarino F. Baker G. (2001). Analysis of amino acids and catecholamines, 5-hydroxytryptamine and their metabolites in brain areas in the rat using *in vivo* microdialysis. Methods (San Diego, Calif.) 23 (1), 11–20. 10.1006/meth.2000.1102 11162146

[B34] Peixoto J. V. Rocha M. G. Nascimento R. T. L. Moreira V. V. Kashiwabara V. V. (2014). Candidíase - Uma Revisão de Literatura. Braz. J. Surg. Clin. Res. 8 (2), 75–82.

[B35] Plock N. Kloft C. (2005). Microdialysis--Theoretical background and recent implementation in applied life-sciences. Eur. J. Pharm. Sci. 25 (1), 1–24. 10.1016/j.ejps.2005.01.017 15854796

[B36] Ray A. Malin D. Nicolau D. P. Wiskirchen D. E. (2015). Antibiotic tissue penetration in diabetic foot infections: a review of the microdialysis literature and needs for future research. J. Am. Podiatric Med. Assoc. 105 (6), 520–531. 10.7547/14-036.1 26667505

[B37] Rottbøll L. A. H. Skovgaard K. Barington K. Jensen H. E. Friis C. (2015). Intrabronchial microdialysis: effects of probe localization on tissue trauma and drug penetration into the pulmonary epithelial lining fluid. Basic and Clin. Pharmacol. and Toxicol. 117 (4), 242–250. 10.1111/bcpt.12403 25827198

[B38] Shippenberg T. S. Thompson A. C. (2001). Overview of microdialysis. Curr. Protoc. Neurosci. Chapter 7, Unit7.1. 10.1002/0471142301.ns0701s00 PMC2538639 18428520

[B39] Tegeder I. Schmidtko A. Bräutigam L. Kirschbaum A. Geisslinger G. Lötsch J. (2002). Tissue distribution of imipenem in critically ill patients. Clin. Pharmacol. Ther. 71 (5), 325–333. 10.1067/mcp.2002.122526 12011818

[B40] Torres B. G. S. Helfer V. E. Bernardes P. M. Macedo A. J. Nielsen E. I. Friberg L. E. (2017). Population pharmacokinetic modeling as a tool to characterize the decrease in ciprofloxacin free interstitial levels caused by Pseudomonas aeruginosa biofilm lung infection in wistar rats. Antimicrob. Agents Chemother. 61 (7), 025533–e2616. 10.1128/AAC.02553-16 PMC5487627 28461311

[B41] Tøttrup M. Hardlei T. F. Bendtsen M. Bue M. Brock B. Fuursted K. (2014). Pharmacokinetics of cefuroxime in porcine cortical and cancellous bone determined by microdialysis. Antimicrob. Agents Chemother. 58 (6), 3200–3205. 10.1128/aac.02438-14 24663019 PMC4068497

[B42] Tsai T.-H. (2003). Assaying protein unbound drugs using microdialysis techniques. J. Chromatogr. B, Anal. Technol. Biomed. Life Sci. 797 (1–2), 161–173. 10.1016/j.jchromb.2003.08.036 14630148

[B43] Walsh T. J. Yeldandi V. McEvoy M. Gonzalez C. Chanock S. Freifeld A. (1998). Safety, tolerance, and pharmacokinetics of a small unilamellar liposomal formulation of amphotericin B (AmBisome) in neutropenic patients. Antimicrob. Agents Chemother. 42 (9), 2391–2398. 10.1128/AAC.42.9.2391 9736569 PMC105839

[B44] Wang X. Shair Mohammad I. Fan L. Zhao Z. Nurunnabi Md Sallam M. A. (2021). Delivery strategies of amphotericin B for invasive fungal infections. Acta Pharm. Sin. B 11 (8), 2585–2604. 10.1016/j.apsb.2021.04.010 34522599 PMC8424280

